# Retrotransposons: A New Frontier in In Vivo Genome Engineering

**DOI:** 10.3390/pharmaceutics18101316

**Published:** 2026-10-16

**Authors:** Philippe Kieffer-Kwon, Meghan Harris, Daniel Getts, Robert Hofmeister

**Affiliations:** Create Medicines, 300 Technology Square, Cambridge, MA 02139, USA

**Keywords:** retrotransposons, non-LTR retrotransposons, in vivo genome engineering, gene integration, genome editing, lipid nanoparticles, CAR-T cells, R2, LINE-1, Vingi-1, L2-2, RTE-1

## Abstract

Genome engineering has transformed cell therapy, but clinical implementation remains largely confined to ex vivo workflows followed by adoptive cell transfer. These approaches require costly manufacturing, often rely on viral vectors, and carry risks associated with unwanted genomic alterations. Recent advances in lipid nanoparticles (LNPs) enable genome-editing components to be delivered directly to specific cell populations in vivo, eliminating the need for ex vivo manufacturing. Several RNA-based genome-editing approaches have entered clinical trials. Nevertheless, current in vivo approaches remain largely limited to gene disruption or small sequence modifications. Conventional integration of large payloads generally requires DNA donors, which can be immunogenic, have limited nuclear access, and pose substantial LNP formulation challenges. When expression of therapeutic proteins such as chimeric antigen receptors (CARs) is required, LNP-based approaches predominantly deliver mRNA, leading to transient expression, whereas many applications require durable expression through stable genomic integration. Retrotransposons offer a solution because they have naturally evolved to reverse-transcribe RNA templates directly into chromosomal DNA. Engineered retrotransposon-derived platforms can integrate multi-kilobase payloads from RNA without an exogenous DNA donor. These platforms combine durable expression with RNA-only delivery, but clinical translation requires safe permanent integration, copy-number control, efficient delivery of all required components, and sufficient activity in disease-relevant primary cells. In this review, we describe the classes of non-LTR retrotransposons; examine the design principles underlying their adaptation as genome-editing tools; and compare emerging platforms derived from R2, LINE-1, Vingi, and RTE elements. These systems support integration strategies ranging from naturally evolved insertion profiles, such as site-specific insertion into ribosomal DNA (rDNA) or dispersed genome-wide integration, to fully programmable insertion. Several retrotransposon-based platforms have demonstrated multi-kilobase payload insertion, functional engineering of primary human T cells, and in vivo activity in mouse or non-human primate models, demonstrating their clinical promise. We also discuss key translational challenges. With continued optimization, retrotransposon-based platforms have the potential to bridge the gap between transient RNA delivery and durable gene writing, establishing a versatile foundation for the next generation of in vivo genetic medicines.

## 1. Introduction

Rapid advances in cell-targeted lipid nanoparticle (tLNP) formulations are enabling genetic medicines to reach specific cell populations directly within the patient. By bypassing the isolation, engineering, expansion, and reinfusion of cells, in vivo approaches have the potential to shorten manufacturing timelines, reduce production costs, improve treatment accessibility and specificity, and spare patients from burdensome conditioning, which often requires cytotoxic conditioning regimes prior to transplantation of ex vivo-derived T cell and HSPC cell therapy medicines. Early clinical studies have begun to validate the feasibility and safety of in vivo genome editing, primarily in the liver. However, the editing outcomes achieved by current clinical programs remain mainly limited to gene inactivation or the correction of small numbers of nucleotides [1,2,3,4,5,6]. Site-specific integration of large therapeutic payloads directly in vivo remains considerably more challenging but, if achieved, enables a broad range of therapeutic applications [7].

Current clinical strategies for targeted gene insertion may require the coordinated delivery of multiple vehicles to accommodate the number and size of their molecular components, including an LNP carrying a programmable nuclease together with an adeno-associated virus (AAV) carrying the donor DNA [8] or a dual-AAV system [9]. In addition to the complexity of manufacturing and administering two delivery vehicles, these approaches retain potential liabilities associated with DNA double-strand breaks (DSBs) and the use of exogenous donor DNA. More recently, hybrid technologies such as PASTE [10] and PASSIGE [11] have combined prime editing with large serine recombinases to enable the targeted insertion of large DNA payloads without introducing a site-specific DSB [12]. However, avoiding nuclease-induced DSBs does not by itself eliminate the safety and delivery challenges associated with exogenous donor DNA. Cytosolic double-stranded DNA can activate innate DNA-sensing pathways, eliciting inflammatory responses and cytotoxicity that may compromise cell viability and editing efficiency. Donor DNA can also be captured at genomic DNA breaks, producing unintended insertions or aberrant on-target products containing donor fragments, concatemeric copies, or vector-backbone sequences. In addition, DNA-dependent insertion requires efficient nuclear delivery of the donor. Although highly versatile, DNA-dependent insertion platforms require efficient nuclear delivery of the donor and coordinated delivery and activity of multiple molecular components, including a Cas nickase, reverse transcriptase, guide RNAs, a recombinase or integrase, and a DNA donor, creating substantial delivery and manufacturing challenges for in vivo therapeutic applications.

Retrotransposons offer an alternative strategy, as they have evolved to copy RNA-encoded genetic information directly into chromosomal DNA [13]. In their native configuration, retrotransposon RNAs encode one or more multifunctional proteins that couple target-DNA recognition and cleavage with reverse transcription of an associated RNA template. The evolution, biochemistry, structural biology, and native target-site selection of retrotransposons fall outside the scope of this review and have recently been comprehensively reviewed elsewhere [14,15,16,17,18,19] and will not be discussed here. Depending on the retrotransposon family [20], integration-site selection can range from highly sequence-specific to broadly dispersed across the genome. Nevertheless, several retrotransposon-derived systems can support the integration of multi-kilobase payloads without requiring delivery of an exogenous DNA donor into the nucleus or a DSB [21,22]. Their highly processive reverse transcriptases can directly copy an RNA-encoded therapeutic cargo into the genome, thereby avoiding the risk of random donor-DNA integration and innate DNA sensing. Following cytoplasmic delivery, the RNA components are translated and assembled into integration-competent ribonucleoprotein complexes capable of accessing and modifying the genome. Importantly, for biotechnology applications, transient expression of the retrotransposon machinery is sufficient to generate stable genomic integration. RNA-based delivery can therefore limit the duration of cellular exposure to the editing machinery relative to DNA-based vectors, potentially reducing risks associated with prolonged editor activity, including cellular toxicity and unintended integration. RNA immunogenicity can be further mitigated through sequence optimization, rigorous purification, and the incorporation of modified nucleosides such as N1-methylpseudouridine when they do not compromise platform activity.

All-RNA retrotransposon-based platforms are therefore particularly well suited for in vivo genome engineering, since the editing machinery and multi-kilobase payload can both be delivered in a transient, LNP-compatible RNA format. This configuration could combine cell-targeted, scalable nonviral administration and transient editor exposure with durable genomic integration of large therapeutic payloads. The growing potential of this approach has stimulated substantial interest across academia and the biotechnology industry, leading to the development of multiple retrotransposon-derived genome-editing platforms, several of which have progressed into advanced preclinical evaluation. In this review, we catalogue and dissect these emerging technologies, compare their molecular architectures, targeting strategies, cargo capacities, editing fidelity, and delivery requirements, and examine both their advantages and current limitations. Together, these advances may lay the foundation for a new generation of programmable in vivo genetic medicines.

## 2. Retrotransposon Clades

Transposons comprise a diverse group of mobile genetic elements that have shaped the evolution of virtually all genomes. They are broadly classified into two major classes. Class II transposons, often referred to as “jumping genes,” are DNA transposons that mobilize through a cut-and-paste mechanism, in which the transposon is excised from its original genomic location and inserted into a new site. Class I transposons, or retrotransposons, propagate through an RNA intermediate by reverse transcribing their own transcripts into DNA before integration into the genome, thereby increasing their copy number.

Retrotransposons are further divided into long terminal repeat (LTR) and non-LTR retrotransposons [23]. LTR retrotransposons are evolutionarily related to retroviruses and rely on a virally derived integrase to insert the newly synthesized cDNA into the host genome [24]. In contrast, non-LTR retrotransposons lack an integrase and instead integrate through target-primed reverse transcription (TPRT), a mechanism initiated by endonuclease-mediated nicking of the target DNA followed by reverse transcription directly at the integration site.

Among non-LTR retrotransposons, long interspersed nuclear elements (LINEs) constitute the principal class of autonomous elements. Typically larger than 6 kb, LINEs encode the proteins required for their own mobilization, including an endonuclease and a reverse transcriptase. In contrast, short interspersed nuclear elements (SINEs) are generally smaller than 1 kb and are non-autonomous, relying on proteins encoded by LINEs to complete their retrotransposition cycle [25].

LINEs are widely distributed throughout eukaryotes and have recently attracted considerable attention as novel platforms for genome engineering and gene therapy. Based on the structural and evolutionary characteristics of their endonuclease domains, LINEs can be divided into two major groups (Figure 1). The ancestral group encodes a restriction enzyme-like endonuclease (RLE), whereas the more recently evolved group encodes an apurinic/apyrimidinic endonuclease (APE). Each group comprises multiple evolutionary clades that have diverged over hundreds of millions of years and have adapted to distinct host species and genomic niches. Consequently, these clades differ in their target-site recognition, integration mechanisms, cargo capacity, reverse transcriptase properties, and host interactions. The RLE group includes the R2, R4, HERO, CRE, and NeSL clades, while the APE group includes the LINE-1, Vingi, RTE, CR1, and Jockey clades, among others. This remarkable evolutionary diversity provides a rich repertoire of naturally evolved molecular machines with distinct biochemical properties that can be exploited for therapeutic genome engineering. Members of both groups have recently emerged as promising platforms for gene insertion, although the greatest progress to date has been achieved with members of the R2 clade of the RLE group and the LINE-1, Vingi, and RTE clades of the APE group.

Throughout this review, retrotransposons are designated using four-letter species abbreviations, comprising the first two letters of the genus and species names, following Zhang et al. [26]. This convention avoids ambiguity associated with two-letter nomenclature; for example, R2Tg could refer to either R2TaGu from Taeniopygia guttata or R2TiGu from Tinamus guttatus (Table 1).

## 3. Design Principles for Retrotransposon-Based Genome-Editing Platforms

### 3.1. Mechanism

At first sight, RLE- and APE-type non-LTR retrotransposons use a common strategy to propagate through the host genome (Figure 2). Once transcribed, the retrotransposon RNA directs the synthesis of one or more proteins, including a multifunctional protein containing endonuclease and reverse transcriptase domains. This multifunctional protein, alone or together with other element-encoded proteins, associates with the retrotransposon RNA to form a ribonucleoprotein complex. The complex then engages the genomic target site, where the endonuclease first cleaves the bottom DNA strand, exposing a 3′-OH that is positioned against the 3′ end of the retrotransposon RNA. This genomic 3′-OH directly primes first-strand cDNA synthesis at the target site through a process known as target-primed reverse transcription (TPRT) [16,19,40]. The opposite DNA strand is subsequently cleaved, although the timing of this second cleavage relative to first-strand synthesis differs among retrotransposons. The resulting genomic 3′-OH can then prime synthesis of the complementary DNA strand using the first-strand cDNA as a template. Formation of this second primer–template junction is thought to involve a still incompletely understood strand-transfer or template-jumping mechanism. The retrotransposon RT then switches from RNA-dependent to DNA-dependent DNA synthesis and extends the second DNA strand. Finally, host DNA-repair factors complete end processing, gap filling, and ligation, thereby resolving the newly synthesized retrotransposon DNA into a stable genomic insertion.

At the molecular level, substantial differences exist between APE- and RLE-type retrotransposons, as well as among members of the same retrotransposon class. These differences can profoundly influence how a retrotransposon is used as a genome-editing tool and determine the engineering adaptations required for gene and cell therapy applications.

### 3.2. Integration Site

APE- and RLE-type retrotransposon proteins have distinctive domain organizations. In APE-type retrotransposons, the endonuclease domain is located at the N-terminus and is followed by the reverse-transcriptase domain. In RLE-type retrotransposons, the reverse-transcriptase domain precedes a C-terminal restriction-like endonuclease domain. RLE proteins also contain zinc finger(s) and Myb DNA-binding domains that contribute to target-site recognition. Consequently, many RLE retrotransposons integrate at specific genomic sites, such as the 28S rRNA gene for R2 or the 18S rRNA gene for R7 [41], whereas APE-type retrotransposons such as LINE-1 and Vingi display much broader target-site distributions [42]. However, site specificity does not eliminate the need to control insertion copy number. The 28S rRNA gene is present in many copies across the ribosomal DNA arrays, and excessive integration into these repeats could still compromise cell fitness, even though the multicopy rDNA arrays have been proposed as potentially permissive integration sites. Consequently, a platform using a site-specific or broadly integrating retrotransposon must rigorously assess and control the risks of unintended or multicopy integration.

### 3.3. Cleavage Pattern

Another important consideration is the position and timing of the DNA nicks generated during retrotransposition. Closely spaced nicks introduced by paired Cas9 nickases have previously been associated with genomic deletions [43]. Retrotransposition is also a complex, multistep process that can be interrupted by abortive reverse transcription, failed strand transfer, or inappropriate template jumping [14]. Consequently, retrotransposon-mediated DNA cleavage can generate TPRT-independent DSBs, as observed with R2TaGu [15,34], which cleaves the two DNA strands with only a 2-nucleotide offset between the nicking sites. LINE-1 uses a larger offset of 8–12 nucleotides between the two nick sites but can nevertheless generate DSBs [15,34]. The use of a wild-type retrotransposon endonuclease therefore requires careful characterization of cleavage kinetics, genomic deletion, chromosomal rearrangements, and other consequences for genome integrity.

### 3.4. Activity and Protein Engineering

Retrotransposons must propagate without catastrophically damaging the host genome. Over evolutionary time, highly active elements have often been suppressed, inactivated, or eliminated. Most retrotransposon sequences present in the human genome are therefore molecular fossils, frequently consisting of truncated or mutated fragments inherited from ancestral species [23]. Consequently, naturally occurring retrotransposons rarely display the level of activity required for therapeutic genome editing. Their activity must generally be improved through rational engineering of the retrotransposon proteins, optimization of the RNA template and its structural elements, or the addition of new functional domains. These modifications must increase integration efficiency without compromising target specificity, genomic integrity, or cellular tolerability.

### 3.5. Ribonucleoprotein Assembly and Delivery Format

The mechanism of retrotransposon ribonucleoprotein assembly can strongly influence the architecture and delivery format of the resulting genome-editing platform. For instance, the ORF2 protein of LINE-1 is unstable at physiological temperature but is markedly stabilized by binding a poly(A) RNA [40], supporting a model in which ORF2p rapidly associates with the poly(A) tail of its own encoding RNA after translation. Additionally, the LINE-1-encoded protein ORF1p further promotes this cis-preferential RNP assembly by forming condensates around the LINE-1 RNA [44]. Maintaining this evolved LINE-1 cis RNA binding preference may therefore require delivery of a single RNA containing ORF1, ORF2, and the therapeutic payload, potentially exceeding 7 kb. Such long RNAs create substantial challenges for RNA synthesis, stability, formulation, and LNP encapsulation. By contrast, R2 proteins recognize structured elements in the R2 3′ UTR through an N-terminal extension of the reverse-transcriptase domain, referred to as NTE-1 in R2BoMo [18] and RT−1 in R2TaGu [15]. This trans recognition allows the protein-coding RNA and the donor RNA to be delivered as separate molecules. Such modularity can simplify system design and reduce the size of individual RNA components. However, the donor RNA must retain the R2 recognition element, and at least part of this sequence is copied into the genome together with the payload. Hence, the resulting retrotransposon-derived genomic scar must be considered when selecting therapeutic applications.

Importantly, the donor RNAs are designed to prevent direct payload expression through reverse payload orientation or upstream sequences that preclude productive translation. Consistent with this design, no payload expression was detected with the donor RNA alone in trans or with a retrotransposon deficient in either reverse transcriptase or endonuclease activity in cis. Reporter expression therefore provides a functional readout of retrotransposition in these assays.

### 3.6. Compatibility with the Human Cellular Environment

The evolutionary origin and host distribution of a retrotransposon may also restrict its use in human cells. Although retrotransposons are found throughout eukaryotes, LINE-1 is the only autonomous non-LTR retrotransposon family currently known to remain active in humans. Most alternative systems being considered for genome engineering originate from non-human species. Before adapting such an element for therapeutic use, it is therefore important to establish whether the host factors required for retrotransposition in the native species are conserved and function similarly in human cells. Conversely, human host factors may either support or restrict a retrotransposon by affecting its intracellular localization, RNP assembly, reverse transcription, integration, or DNA-repair outcomes. For example, human Polθ promotes microhomology-mediated joining of R2-derived cDNA and thereby influences insertion completeness and junction precision, whereas the 53BP1-shieldin-CST-Polα pathway preferentially supports severely truncated insertions [45]. In LINE-1, TREX1 restricts retrotransposition by binding ORF1p and altering its intracellular localization, whereas PABPC1 binding to the poly(A) tail promotes efficient RNP assembly [46,47].

### 3.7. Immune Response

Finally, the expression of non-human retrotransposon proteins must be evaluated for cellular tolerability and immunogenicity. Foreign proteins and RNA structures may activate innate immune pathways, while prolonged or repeated expression could induce adaptive immune responses. These considerations are particularly important for in vivo engineering, where systemic exposure, tissue distribution, and immune surveillance are more difficult to control than in ex vivo cell-manufacturing workflows. For example, LNP-mediated delivery may expose the liver, spleen, and antigen-presenting cells to RNA and non-human retrotransposon proteins, potentially triggering innate inflammation or editor-specific adaptive immune responses. Immunogenicity should therefore be assessed using a tiered approach combining human primary-cell assays with immunocompetent in vivo studies evaluating cytokine induction, anti-editor antibodies, editor-specific T-cell responses, tissue inflammation, and the persistence of edited cells [48].

### 3.8. Information Sources and Limitations

This review draws on peer-reviewed research articles, patent applications, and conference presentations. For several industrial platforms, publicly available information comes primarily from patent and conference disclosures, which do not undergo the same scientific peer-review process as full research articles. Patent applications are typically published approximately 18 months after their earliest priority date, and publicly available information may therefore not reflect the latest developments in platform design. Describing these platforms often requires combining information from multiple sources that may refer to different configurations or stages of development. Accordingly, the descriptions presented here reflect our synthesis of the available disclosures and may not fully represent the systems currently being developed in industry. Substantial differences in experimental conditions and incomplete disclosure of results make direct comparisons between platforms difficult and potentially misleading. Given the rapid pace of retrotransposon platform development, such comparisons may also quickly become outdated. Nevertheless, Supplementary Table S1 summarizes a representative selection of experimental data reported in patent applications for the platforms discussed in the following sections.

## 4. RLE-Based Platforms

Among RLE-derived genome-integration systems being developed for therapeutic applications, R2 has emerged as the most widely exploited scaffold (Figure 3A). R2 retrotransposons are broadly distributed across metazoans but are absent from mammals, whereas their highly conserved insertion site within the 28S rRNA gene remains present in humans. The Bombyx mori R2 element (R2BoMo) is by far the most characterized member of this family; however, several groups have selected R2 proteins from other phylogenetic subclades because they possess properties that may be better suited to genome engineering. Native R2 RNA comprises a single open reading frame (ORF) flanked by 5′ and 3′ untranslated regions (UTRs). The 5′ UTR contains an HDV-like self-cleaving ribozyme with a double-pseudoknot fold that processes the R2 RNA from the 28S rRNA cotranscript [17]. In many R2 lineages, cleavage occurs within the upstream 28S sequence, leaving a short 5′ rDNA extension that can facilitate formation of the upstream integration junction. The ORF encodes a multifunctional protein containing sequence-specific DNA-binding, reverse transcriptase, and endonuclease domains, whereas the structured 3′ UTR mediates recognition of the RNA template by the R2 protein. In some R2 systems, a short terminal sequence homologous to the rDNA immediately downstream of the nick further promotes RNA-DNA annealing and initiation of homology-mediated TPRT [15].

### 4.1. Precise RNA-Mediated Insertion of Transgenes (PRINT) by Addition Therapeutics

Developed in Kathleen Collins’s laboratory, precise RNA-mediated insertion of transgenes (PRINT) is a chimeric engineered approach derived from the R2 retroelement family for targeted integration at the 28S rRNA integration site. PRINT uses avian R2 elements from the R2-A3 clade, initially including R2GeFo, R2TaGu, R2ZoAl, and R2TiGu [26]. R2TaGu and R2ZoAl proteins displayed the highest cellular insertion activity, whereas the R2GeFo and R2ZoAl 3′ UTRs served as efficient template-recognition modules. PRINT is a two-RNA platform in which one mRNA encodes the R2 protein and a separate template RNA carries the transgene expression cassette. The original template RNA begins with a Tribolium castaneum R2-derived 5′ module, termed TCA5, that contains an HDV-like self-cleaving ribozyme. Self-cleavage generates a mature RNA bearing 28 nucleotides of homology to the rDNA immediately upstream of the first nick, while the stable double-pseudoknot ribozyme fold provides cap-like protection against 5′ exonucleolytic degradation. The template then contains the transgene expression cassette, originally demonstrated with GFP, followed by an avian R2 3′ UTR, a four-nucleotide sequence homologous to the rDNA immediately downstream of the nick (R4), and a tract of 22 adenosines (A22). In RPE cells, PRINT-mediated integration of a GFP expression cassette into 28S rDNA generated approximately 35% GFP-positive cells 24 h after lipofection, increasing to approximately 50% when uridine was replaced with N1-methylpseudouridine (m1Ψ) in the template RNA. In subsequent constructs, an HDV genomic-strand ribozyme (HDV-gRZ) was used in place of the TCA5 5′ module, while the 3′ end was optimized by engineering its R4-A-rich tail and GeFo-derived 3′ UTR. The best variants increased reporter-based insertion activity by up to approximately 1.9-fold relative to the corresponding control templates [30,31].

Accordingly, co-delivery of the two PRINT RNAs using wild-type R2ZoAl initially generated approximately 45% GFP-positive RPE cells, with sorted GFP-positive cells carrying an average of approximately 33 total insertions. However, the GFP-positive fraction declined to below 2% after 14 days of continuous culture. To control the insertion load, the Collins laboratory introduced the R1119A and R1103A substitutions into R2TaGu and R2ZoAl, respectively, generating endonuclease-tuned (ENT) variants [26]. R2ZoAl-R1103A reduced the average copy number to approximately 2.5 insertions per GFP-positive cell. Both ENT variants stabilized the edited populations, with R2ZoAl-ENT maintaining approximately 6–8% GFP-positive cells over 20 days.

PRINT is now being developed by Addition Therapeutics, which has reported promising preclinical proof-of-concept studies. These include production of the HIV-neutralizing eCD4-Ig biologic by primary human hepatocytes and skeletal muscle cells in vitro and following in vivo delivery in rats, as well as functional production of two anti-VEGF biologics by RPE cells in vitro. In a Fabry disease mouse model, a single LNP dose produced durable, supraphysiological plasma α-galactosidase A activity, which sustained an approximately 80 to 90% reduction in plasma lyso-Gb3 for 16 weeks and reduced kidney and heart lyso-Gb3 by approximately 80% at the end of the study. Following a single LNP dose in NHPs, PRINT-mediated hepatic integration of a human erythropoietin (hEPO) cassette produced stable serum hEPO levels of approximately 10 to 30 ng/mL throughout the 20-week study [32]. Together, these findings illustrate that all-RNA retrotransposon-based platforms are particularly well suited to in vivo genome engineering.

### 4.2. En-R2Tg System by the Institute of Zoology, Beijing

Derived from the R2TaGu retrotransposon, the en-R2Tg platform was established in Wei Li’s laboratory as a two-RNA system for targeted integration at the 28S rRNA integration site [33]. One RNA encodes an optimized R2TaGu protein (R2Tgopt) bearing nuclear localization signals at both the N and C termini. From 5’ to 3’, the second RNA comprises a 100 nt homology arm, the R2TaGu 5’ UTR containing its characteristic HDV-like ribozyme, the payload, and the R2TaGu 3’ UTR followed by a 100 nt downstream homology arm. Self-cleavage by the ribozyme generates a mature donor RNA retaining 28 nt of 5’ homology. Wild-type R2TaGu initially achieved approximately 2% GFP integration in HEK293T cells.

To improve its activity, Li and colleagues used structure- and sequence-guided engineering to modify residues predicted to interact with the RNA-DNA hybrid or the target rDNA and to restore conserved residues found in the RT domains of related R2 proteins from the same clade. Iterative screening identified a combination of three beneficial substitutions: A793V, Q955R, and G1098R. In addition, two chromatin-modulating peptides were incorporated into R2Tgopt, one at the N-terminus and the other within an internal linker upstream of the RT domain. In parallel, the donor RNA was optimized by deleting a large stem-loop structure from the R2TaGu 3’ UTR, generating a shortened donor designated donoropt. Interestingly, as observed with the PRINT platform, incorporation of N1-methylpseudouridine (m1Ψ) into the donor RNA further improved platform activity. Together, these optimizations produced an integration efficiency of 24.8% in Huh7 cells following LNP delivery and maintained efficiencies above 10% with donor sequences of up to 2.5 kb. Moreover, microinjection of the en-R2Tg RNAs into the pronucleus of mouse zygotes yielded more than 60% targeted GFP integration.

However, when applied to primary T cells in vitro, en-R2Tg achieved an integration efficiency below 1% four days after nucleofection. This sharp reduction highlights the importance of optimizing retrotransposon-based platforms directly in therapeutically relevant primary cells rather than relying on their performance in transformed cell lines. It may also reflect differences in the host factors required to complete payload integration.

### 4.3. Minimod Donor by the Massachusetts Institute of Technology

Another R2TaGu-based platform was developed in Feng Zhang’s laboratory for targeted integration at the 28S rRNA integration site [15]. Structural and biochemical characterization of R2TaGu, including cryo-electron microscopy of the complex initiating TPRT, enabled the rational design of a compact all-RNA system. Taking advantage of the ability of R2TaGu to act in trans, the platform uses separate editor and donor RNAs. The editor RNA encodes a human-codon-optimized R2TaGu protein flanked by UTRs adapted from the BNT162b2 vaccine and is synthesized as a capped, m1Ψ-containing, and polyadenylated mRNA.

The donor RNA was extensively minimized. The entire R2TaGu 5′ UTR, including its HDV-like ribozyme, was removed and replaced with a degradation-resistant 5′ modification comprising a locked nucleic acid cap analog followed by five 2′-O-methyl-modified nucleotides. Together with the remaining homology sequence, these nucleotides form a 42 nt 5′ homology arm, followed by the payload, 100 nucleotides of the truncated R2TaGu 3′ UTR, a 5 nt 3′ homology arm, and a 40 nt poly(A) tail. As observed with other R2-derived platforms, incorporation of m1Ψ into the donor increased integration efficiency.

When the editor and miniMod donor RNAs were co-encapsulated in SM-102 LNPs, functional GFP integration was detected in more than 90% of HEK293FT cells approximately 44 h after transfection. At this early time point, ddPCR combined with long-read sequencing estimated approximately 3.3 full-length transgene copies per genome, indicating frequent multicopy integration. Over the following days, however, the GFP-positive fraction decreased by as much as threefold under some conditions and was accompanied by a decline in GFP fluorescence intensity. GFP-positive cells proliferated more slowly than GFP-negative cells, indicating progressive counterselection of highly edited cells. The authors propose that both multiple integrations within the 28S rDNA arrays and TPRT-independent DNA double-strand breaks generated by R2TaGu may contribute to this fitness defect. Changing the editor-to-donor weight ratio in the LNPs from 1:1 to 1:4 reduced editor exposure while maintaining similar initial integration levels and improved transgene persistence, with approximately 40% of cells remaining GFP-positive after 20 days.

The miniMod platform also remained active in post-mitotic myotubes and in HEK293FT cells maintained in early S phase with aphidicolin, although integration was less efficient in aphidicolin-treated cells than in untreated cells. Thus, active cell division enhances R2TaGu-mediated integration but is not strictly required. Nevertheless, miniMod achieved less than 1% integration in primary human T cells, further illustrating that translating retrotransposon-based technologies from permissive cell lines to primary cells requires substantial optimization.

### 4.4. Site-Specific Target-Primed Insertion Through Targeted CRISPR Homing of Retroelements (STITCHR) by the Massachusetts Institute of Technology

The STITCHR approach fuses R2 elements together with a CRISPR-Cas9 nickase, enabling programmable cargo integration. The two-RNA platform STITCHR emerged from mechanistic studies of R2TaGu showing that its RT and RLE activities can complement in trans and that internal homology arms matching the intended genomic insertion site can redirect TPRT away from the native 28S rDNA target [34]. In particular, an RNA donor comprising a payload flanked by 50 nt homology arms, a minimal 15 nt element of the 5′ UTR, and no 3′ UTR enabled retargeting of R2-mediated integration and outperformed the full-UTR donor in biochemical assays.

Fell et al. subsequently surveyed single-ORF RLE retrotransposons identified by their characteristic C-terminal RT and RLE domain architecture and selected R2TaOc (published as R2Tocc). Although this element was recovered from a contaminant scaffold in the Talpa occidentalis genome assembly, phylogenetic analysis suggests that it is probably of avian origin. R2TaOc combined efficient retargeting with minimal background insertion at its native 28S rDNA site, making it particularly suitable for engineering. The resulting STITCHR editor comprises SpCas9H840A fused through an XTEN linker to the N-terminus of R2TaOcΔ1–169 and contains five nuclear localization signals distributed across the fusion protein.

Together with target-matched donor RNA and one or two sgRNAs, this editor enabled integration at several endogenous loci, including AAVS1, NOLC1, LMNB1, and EMX1. At NOLC1, paired nCas9-induced nicks enhanced integration compared with either guide alone, whereas the top-strand-nicking guide alone was approximately fourfold more effective than the bottom-strand-nicking guide. Nevertheless, the native R2TaOc RLE activity remained necessary for efficient integration, and the precise coordination between the Cas9 and R2 nuclease activities remains unresolved. Similarly, how R2TaOc recruits a 3′-UTR-free donor retaining only the minimal 15 nt 5′ element, rather than the structured 3′-UTR recognition element characterized in other R2 orthologues, has not been defined. In HEK293FT cells, STITCHR supported scarless insertion of a 12.7-kb payload at NOLC1 with approximately 9% junction-positive editing. PacBio HiFi sequencing showed that 99.92% of recovered insertion reads represented complete integrations, with only 0.08% incomplete integrations and no detectable concatemers. Two-vector adenoviral delivery achieved approximately 6% integration in HEK293FT cells but only approximately 0.5% precise integration in quiescent primary human hepatocytes. Although still at an early stage of development, STITCHR provides an important proof of principle for redirecting R2-mediated integration beyond rDNA and demonstrates flexibility across several target loci, homology-arm lengths, and guide configurations.

### 4.5. R2 Helpers by Typewriter Therapeutics

Building on the extensive R2 expertise accumulated in Haruhiko Fujiwara’s laboratory at the University of Tokyo [49,50,51], Typewriter Therapeutics aims to develop the therapeutic potential of R2 retrotransposons for targeted integration at the 28S rRNA integration site. The company has not yet publicly disclosed a named development candidate or supporting preclinical package. However, its patent portfolio highlights two avian R2 orthologs: R2AcGe, derived from the Eurasian goshawk Accipiter gentilis, and R2MiRu, derived from the barred forest falcon Micrastur ruficollis, designated R2Ag and R2MicRuf, respectively, in the patent applications [27,28]. In a trans RNA format, a helper mRNA encoding the R2 protein was combined with a donor RNA carrying a GFP cassette flanked by the cognate R2 UTRs. The R2AcGe donor additionally contained a downstream 28S-rDNA homology sequence, although its precise length and sequence were not disclosed, whereas the presence of an analogous sequence in the R2MiRu donor was not specified. Most of the R2AcGe 3’ UTR could be removed while retaining a functional 70 nt core. In separate HEK293T lipofection experiments, R2AcGe and R2MiRu yielded 24.5% and approximately 12% GFP-positive cells, respectively. For R2AcGe, S-EPTS/LM-PCR [52] indicated that more than 99.5% of integration events occurred at the intended 28S-rDNA target. R2AcGe also generated 1.1% and 0.56% GFP-positive cells in primary human T cells and hepatocytes, respectively. A separate patent application showed that HEK293T cells could be repeatedly exposed to undisclosed R2 systems, producing cumulative reporter integration [29]. Sequential or concurrent delivery of GFP and mCherry generated a small population of double-positive cells, providing functional evidence of dual payload integration within individual cells.

## 5. APE-Based Platforms

APE-type non-LTR retrotransposons encompass diverse lineages, including LINE-1, L2, Vingi, RTE, CR1, and Jockey. Each family member encodes a multifunctional protein composed of an N-terminal apurinic/apyrimidinic endonuclease followed by a reverse transcriptase, an organization distinct from the C-terminal endonuclease of RLE elements. The number of ORFs encoded by APE-type retrotransposons varies among lineages. LINE-1, the only autonomous retrotransposon currently active in humans, encodes two proteins, namely ORF1p, an RNA-binding chaperone, and ORF2p, which carries endonuclease and reverse-transcriptase activities, whereas the L2-2, Vingi, and RTE elements developed as gene-integration tools encode a single multifunctional protein. During TPRT, the APE nicks the target DNA to expose a 3′ hydroxyl that primes reverse transcription of the associated RNA. In LINE-1, annealing of the RNA poly(A) tail to a T-rich target sequence facilitates this process [53]. Unlike the pronounced site specificity of R2, many APE-type retrotransposons display broad genomic insertion distributions. This relaxed target-site requirement expands the range of accessible genomic contexts but makes genome-wide insertion profiling, copy-number control, and characterization of integration completeness and junction fidelity essential for therapeutic development (Figure 3B).

### 5.1. Driver by Averna Therapeutics

Averna Therapeutics, formerly Exsilio Therapeutics, has disclosed two gene-integration platforms based on APE-type non-LTR retrotransposons for dispersed genome-wide integration. The first uses the zebrafish (Danio rerio) L2-2 element (ZfL2-2; designated here L2-2DaRe), whereas the second uses Vingi-1 from the green anole Anolis carolinensis (published as Vingi-1_Acar and designated here Vingi-1AnCa) [36,37].

L2-2 is a naturally occurring single-ORF retrotransposon encoding a multifunctional protein with APE endonuclease and reverse transcriptase activities. Among the numerous variants evaluated, one of the most extensively engineered L2-2DaRe proteins combined the beneficial substitutions D64K in the endonuclease domain, I343K in a putative RNA-binding region, and N647K and L825G in the reverse transcriptase domain. The protein was further extended with the nucleosome-binding protein HMGN1 at its N-terminus and fused at its C-terminus to an HSV-1 UL12-derived DNA-repair polypeptide followed by the nucleosome-binding protein HMGB1. The driver RNA provides the L2-2DaRe protein in trans to a separate reporter RNA. The reporter RNA contains an inverted CMV-GFP cassette followed by the L2-2DaRe 3′ UTR but lacks the L2-2DaRe 5′ UTR. This system generated approximately 30% GFP-positive U2OS cells 24 h after transfection. However, this early fluorescence readout did not establish stable genomic integration, and integration copy number, junction fidelity, and insertion-site distribution were not disclosed.

Vingi-1 is a compact, single-ORF APE-type non-LTR retrotransposon belonging to the Vingi clade, a sister clade to Ingi [42]. Unlike Ingi elements, Vingi-1 lacks an RNase H domain. The Vingi-1AnCa platform similarly adopts a dual-RNA configuration. The driver RNA unusually retains the native Vingi-1 5′ UTR upstream of the Vingi-1 ORF and terminates with a segmented poly(A) tail arranged as A32N8A74. Separate donor RNAs contain an inverted promoter–payload–polyadenylation signal cassette flanked by Vingi-derived 5′ and 3′ UTRs. Payloads included anti-CD19 CARs and several TCR-subunit fusion receptors structurally equivalent to TRuCs [54], expressed from promoters including EF1α and MNDopt. Following LNP delivery to activated primary human T cells, the platform generated up to 59.3% CAR-positive cells at day 12. Under this condition, bulk dPCR detected 137.2 CAR copies per 100 genomes, corresponding to an estimated average of 2.3 copies per CAR-positive cell. However, donor-dependent estimates varied substantially and exceeded 10 copies per CAR-positive cell in some cases. The resulting CAR- and TRuC-expressing T cells mediated NALM6-Luc killing in vitro, and ex vivo-generated CD3γ-TRuC T cells engineered with Vingi-1AnCa suppressed NALM6-Luc leukemia following adoptive transfer into mice. Although these findings demonstrate efficient generation of functional engineered primary T cells, the non-site-specific integration of L2-2DaRe and Vingi-1AnCa, together with variable and sometimes high copy-number estimates, creates a distinct safety profile from site-specific approaches and will require genome-wide insertion-site profiling, comprehensive junction analysis, and single-cell integration copy-number analysis to assess the risks of gene disruption, aberrant transcription, and truncated or chimeric products before clinical translation.

### 5.2. Gene Writer by Tessera Therapeutics

Like Averna Therapeutics, Tessera Therapeutics developed platforms derived from the APE-type non-LTR retrotransposons Vingi-1AnCa (published as Vingi-1_Acar) and RTE-1MoDo from Monodelphis domestica (published as RTE-1_MD) for dispersed genome-wide integration [55]. Both operate in trans using a Writer mRNA encoding a gene-modifying protein containing endonuclease and reverse transcriptase domains and a separate template RNA carrying the payload between cognate retrotransposon 5′ and 3′ UTRs.

S-EPTS/LM-PCR [52] mapped 5055 Vingi-1AnCa insertion sites in iPSCs, of which 43% were intergenic and 47% intronic. This distribution showed less genic bias than historical HIV-1 integration-site data from T-cell latency models [38]. At 24 h after electroporation of T cells or iPSCs, no detectable increase in phosphorylated p53 or p21 was observed with Vingi-1AnCa or RTE-1MoDo, although writer abundance was not measured. Vingi-1AnCa also generated up to 35% GFP-positive T cells without detectable induction of IFNB1 or CXCL10. Together, these results support a weak innate response profile for APE-type non-LTR retrotransposon systems in human cells.

RTE-1MoDo generally produced higher CAR-positive fractions than Vingi-1AnCa. It generated 12 to 15% BCMA-CAR-positive T cells in one study and approximately 15 to 20% in another, depending on the driver configuration. RTE-1MoDo also produced approximately 9% CD20-CAR-positive T cells in vitro. By comparison, Vingi-1AnCa yielded approximately 0.5–2.5% BCMA-CAR-positive cells. However, these experiments differed substantially in template–driver ratios, RNA concentrations, writer configurations, CAR sequences, cell donors, electroporation conditions, and promoters, making direct comparisons difficult. In particular, the best RTE-1MoDo experiment used a 200:1 template–driver ratio, compared with 2:1 in the main Vingi-1AnCa studies. RTE-1MoDo UTRs were subsequently optimized through ancestral sequence reconstruction and mutational screening, increasing GFP-positive primary human T cells from approximately 8% with the wild-type UTRs to 14% following RNA nucleofection [39]. This improvement highlights the potential for further rational optimization of the platform. Moreover, after enrichment, Vingi-1AnCa-engineered BCMA CAR-T cells cleared established RPMI-8226 tumors in four of five NSG xenograft mice and substantially reduced the remaining tumor.

At ASGCT 2026, Tessera presented an undisclosed trans-acting non-LTR Gene Writer delivered using T-cell-targeted LNPs [32]. In a PBMC-engrafted NSG tumor model, a single intravenous dose generated approximately 24% CD19-CAR-positive T cells around day 12, followed by contraction, and controlled tumor burden. In humanized mice, CD20 CAR-T cells induced near-complete B-cell depletion at doses as low as 0.03 mg/kg. In cynomolgus and rhesus macaques, respective doses of 0.1 and 0.05 mg/kg produced peaks of approximately 60% CAR-positive T cells around days 8–12, followed by rapid contraction, while B-cell depletion persisted in blood and lymph nodes for 29 to 35 days. Bulk dPCR values corresponded to an estimated 1.5/2 CAR copies per CAR-positive cell, and CAR expression was observed in naïve and central-memory subsets. These highly encouraging results demonstrate permanent CAR integration in nonhuman primates using an all-RNA retrotransposon-based platform. Given the non-targeted nature of payload integration, characterization of genome-wide insertion sites, junction integrity, and clonal expansion profiles of edited cells will help support the platform’s progression toward clinical translation.

### 5.3. RetroT by CREATE Medicines

Evolved from the CRISPR-Enabled Autonomous Transposable Element (CREATE) platform [22], RetroT harnesses the natural integration machinery of the LINE-1 (L1) retrotransposon together with CRISPR Cas9 nickase for programmable gene insertion in human cells. L1 is the only autonomous retrotransposon family that remains active in humans. Its RNA encodes two proteins: ORF1p, an RNA-binding protein with nucleic acid chaperone activity, and ORF2p, a multifunctional protein with endonuclease and reverse transcriptase activities. Both proteins exhibit a strong cis preference for the RNA from which they are translated. Accordingly, RetroT uses a cis configuration in which the editor proteins and payload are encoded by a single RNA, without requiring the native retrotransposon UTRs for ribonucleoprotein complex formation or efficient payload integration.

The RetroT RNA contains a 5′ CleanCap structure followed by human codon-optimized ORF1 and ORF2 coding sequences separated by an internal ribosome entry site (IRES). Importantly, the ORF2p endonuclease domain is inactivated by the D205A mutation (dEN), thereby preventing ORF2p-mediated random DNA cleavage. The payload is positioned downstream of the ORFs in the reverse orientation and is flanked by two 75 nt primer-binding sites (PBSs) homologous to sequences surrounding the intended genomic integration site. RetroT RNA is co-delivered with SpCas9(H840A) nickase and two guide RNAs, which generate a single-stranded DNA flap bearing a free 3′-hydroxyl group. Annealing of the 3’ PBS to this flap initiates target-primed reverse transcription (TPRT), followed by template switching through the 5’ PBS to complete site-specific payload integration.

In primary human T cells, RetroT achieved up to 12% CD19 CAR-positive cells 12 days after electroporation. Transgene copy-number analysis detected an average of one to two copies per CAR-positive cell, consistent with integration being limited to one or both target alleles. Next-generation sequencing detected no indels or genomic scars at either junction between the integrated CAR transgene and the chromosomal DNA. Notably, replacing SpCas9(H840A) with SpCas9(D10A) or wild-type SpCas9 did not support productive integration, demonstrating the requirement for a correctly oriented single-strand nick and supporting a TPRT-mediated mechanism rather than homology-directed repair in T cells, while whole-genome sequencing of RetroT-edited HEK293T cells identified no off-target integrations.

RetroT-generated CD19 CAR-T cells exhibited antigen-specific cytotoxicity in vitro, efficiently killing CD19-positive NALM-6 cells while sparing CD19-knockout NALM-6 cells across effector-to-target ratios ranging from 1:10 to 1:2. Furthermore, adoptive transfer of a single dose of 10^5^ RetroT-engineered CAR-T cells robustly inhibited tumor growth in an NALM-6 xenograft model established in NSG MHC-I/II double-knockout mice [35].

## 6. Features and Limitations of Retrotransposon-Based Genome Editing

After only a few years of development, retrotransposon-based genome-editing platforms have already demonstrated encouraging performance, with some achieving successful in vivo editing in mouse and nonhuman primate models. These early achievements provide a strong foundation for further refinement and highlight several design principles that may help realize the full potential of these technologies in clinical applications.

### 6.1. Full-Length Integration

A major limitation of retrotransposon-based platforms is the frequent generation of truncated integrations through premature termination of reverse transcription. Among the platforms for which integration completeness has been quantified, R2-based platforms have generally shown truncation rates approaching 50% [15,26,27,33], with the notable exception of STITCHR, for which more than 99.9% of integrations were full length [34]. Among APE-based platforms, approximately 25% of integrations generated by the Vingi-1AnCa-based Driver platform were estimated to be truncated [36]. Truncation frequencies for integrations mediated by the Vingi-1AnCa-based Writer, L2-2DaRe, and RTE-1MoDo platforms have not been publicly disclosed. Like STITCHR, the RetroT platform has demonstrated similarly high payload integrity, with more than 99.9% of integrations being full length [35].

Truncated products are particularly concerning for APE-type platforms that integrate throughout the genome. For example, if only half of the integrations are complete, an average of two full-length copies per edited cell would correspond to approximately four total insertion sites, with the remaining two containing incomplete transgene fragments. These additional insertions could disrupt coding or regulatory regions or, depending on their position and orientation, generate fusion transcripts and potentially chimeric or aberrant proteins.

For R2-based platforms targeting the 28S rDNA arrays, truncated integration presents the risk of inserting a promoter without the complete therapeutic payload into an rDNA repeat. To mitigate this possibility, Addition Therapeutics positioned the promoter at the 5′ end of the PRINT donor RNA, causing it to be copied last during TPRT. A prematurely terminated PRINT integration would therefore generally lack a functional promoter and would be expected primarily to disrupt the targeted rDNA repeat. Although cells contain hundreds of rDNA copies, the cumulative consequences of disrupting multiple repeats remain important when high integration copy numbers are generated.

Interestingly, STITCHR and CREATE-RetroT each achieved reported complete integration rates above 99.9% under the conditions examined despite being derived from different retrotransposon lineages. These observations suggest that integration completeness depends on factors beyond intrinsic reverse-transcriptase processivity. Earlier models of native retrotransposition proposed roles for RNA-template degradation and incidental microhomology in the formation of truncated insertions [56,57]. However, the high completeness reported for STITCHR and CREATE-RetroT raises the possibility that these factors are not the dominant constraints in these engineered systems, highlighting the potential importance of priming architecture and integration-completion mechanisms. Both platforms use Cas9 nickase to generate defined DNA primers and incorporate extensive target-complementary sequences, including homology arms for STITCHR and primer-binding sites for RetroT. These shared features could favor productive TPRT initiation and integration completion. Biochemical studies of R2 reverse transcriptase further indicate that short complementarity between the nascent cDNA terminus and an acceptor RNA can facilitate end-to-end template jumping [58]. These findings provide a rationale for investigating whether engineered target-complementary sequences similarly facilitate template transfer to genomic DNA during integration completion. Recent work on R2 further demonstrated that alternative cellular DNA-repair pathways support intact or truncated insertions, directly implicating host repair in determining integration completeness [45]. Donor architecture, RNA-template integrity, priming geometry, reverse-transcriptase processivity, template switching, and DNA-repair pathway choice are therefore all likely to influence whether an integration is completed. Dissecting these contributions will require complementary approaches, including controlled comparisons of RNA-template integrity, donor architecture, and priming geometry; biochemical assays of reverse-transcriptase processivity and template switching; and perturbation of host DNA-repair pathways. Sequencing of integration products would then reveal how these interventions affect payload completeness and junction structure.

### 6.2. Genotoxicity

Precise control of integration copy number will be critical for the clinical development of retrotransposon-based platforms. This requirement extends beyond functional transgene copies: copy-number measurements should account for both complete and truncated integrations, whereas junction and structural analyses should assess concatemers, inversions, deletions, and other complex byproducts.

Non-LTR retrotransposition can introduce sequence alterations at integration junctions, including non-templated nucleotides, microhomologies, target-site duplications or deletions, and, less frequently, inversions or larger genomic rearrangements [58,59,60,61]. Moreover, full-length products generated by platforms that retain retrotransposon UTRs within the donor RNA necessarily integrate these nontherapeutic sequences together with the payload. Such outcomes increase the importance of either directing integration to genomic safe harbors or characterizing the consequences of integration at other genomic locations.

R2 proteins cleave both strands of their rDNA target in close proximity and can generate TPRT-independent DSBs [15,34]. Because rDNA genes are organized into repetitive arrays across five chromosomes, excessive R2 activity could produce multiple lesions within these arrays, potentially promoting recombination, chromosomal instability, or loss of cellular fitness. Both multiple integrations within the rDNA arrays and TPRT-independent cleavage have been proposed to contribute to the reduced persistence of cells exposed to high R2 editor concentration [15,26]. These observations emphasize that integration-site specificity alone does not establish a superior safety profile. Editor exposure and integration efficiency must therefore be balanced rather than simply maximizing the initial percentage of edited cells.

Although R2-derived systems are highly selective for their canonical 28S rDNA target, rare integrations outside this locus have been detected at genomic sites sharing sequence similarity with the rDNA target [15,26,28,33]. In contrast, broadly integrating APE-type platforms, such as those derived from Vingi-1 and RTE-1, could disrupt essential or tumor suppressor genes, interfere with neighboring gene expression, or activate proto-oncogenes. Genome-wide integration-site mapping is therefore essential, but the absence of detectable off-target integrations by WGS should be interpreted in accordance with sequencing depth, sampling, and analytical sensitivity. Integration-site enrichment methods, such as shearing extension primer tag selection/ligation-mediated PCR (S-EPTS/LM-PCR) [52], can improve recovery of payload–genome junctions. However, detection requires retention of the transgene sequences targeted by the enrichment and amplification primers and remains subject to amplification bias, sampling limitations, and ambiguous mapping in repetitive regions. Junction sequencing alone does not establish full-length payload integrity. Furthermore, for platforms targeting a defined genomic site, identical integration junctions cannot distinguish independent editing events from expansion of a single edited clone.

A separate concern arises from the ability of LINE-1 ORF2p to mobilize RNAs other than its encoding L1 transcript. Despite its strong cis preference, ORF2p can act in trans on nonautonomous retroelements such as Alu and on cellular mRNAs, leading to their stable integration into the genome [62]. No trans-mobilization of endogenous cellular RNAs has been reported following editing with engineered retrotransposon platforms, but the limited number of genome-wide analyses performed to date remains insufficient to exclude rare events. In addition, LINE-1 ORF2p can initiate noncanonical reverse transcription in the cytoplasm and generate RNA:DNA hybrids capable of activating innate immune signaling [40], although these cytosolic products have not been shown to integrate into the genome. Comparable cytosolic priming has not been demonstrated for R2 proteins, although purified R2BoMo RT can initiate reverse transcription in vitro using one RNA molecule to prime cDNA synthesis from another RNA template [63].

Consequently, genotoxicity assessment should combine genome-wide integration-site mapping with measurement of integration copy number, characterization of complete insertion structure, and analysis of genomic rearrangements, including large deletions, inversions, and translocations. This assessment should also cover rearrangements that do not retain payload sequences and, for rDNA-targeted systems, effects on rDNA integrity and transcription. Analyses should be performed in therapeutically relevant cells, with assay detection limits and unresolved genomic regions clearly reported.

### 6.3. Cis and Trans RNA Architectures

Cis and trans configurations differ in how editor production is coupled to donor availability. In cis-configured systems such as RetroT, the retrotransposon coding sequences and therapeutic payload are carried by the same RNA. This architecture exploits the preference of LINE-1 proteins for their encoding RNA and may favor RNP assembly near the site of translation, reducing the requirement for newly synthesized proteins to encounter a separate donor molecule. This proposed kinetic advantage is supported by the cis-preferential activity of LINE-1 proteins, although its contribution to editing efficiency requires platform-specific evaluation.

In trans-configured systems, exemplified by PRINT, the editor and donor are supplied as separate RNAs. This separation permits independent adjustment of their sequences and relative doses but requires sufficient temporal overlap between editor protein availability and an intact donor RNA pool. RNA stability and translation efficiency should therefore be distinguished from RNP assembly efficiency: cis-preferential binding does not itself establish a longer RNA half-life or greater protein production. Trans architectures offer opportunities to optimize editor translation and donor persistence independently, whereas cis architectures require these properties to be balanced within a shared transcript.

Delivery and manufacturing introduce additional trade-offs. Cis architectures reduce the number of retrotransposon RNA species but combine the coding sequences and payload into a longer molecule, increasing the importance of full-length RNA synthesis, purification, stability, and formulation. Trans architectures can reduce individual transcript lengths and permit separate process optimization while introducing additional requirements for component characterization, control of RNA ratios, and effective delivery to the same cells. Neither architecture should be assumed to offer universally superior delivery or manufacturability.

In direct comparisons with unmodified RNA, incorporation of Ψ or m1Ψ increased retrotransposition in T cells for several platforms. This improvement may not be explained solely by reduced innate immune sensing, particularly because unmodified mRNA does not trigger a detectable innate immune response in activated primary human T cells [64], consistent with Tessera Therapeutics’ report of no IFNB1 or CXCL10 induction following delivery of unmodified retrotransposon RNAs to T cells. Enhanced RNA stability, translation, or temporal overlap between editor protein production and donor availability may also contribute to the benefit of Ψ and m1Ψ in trans-configured retrotransposon systems [65,66,67]. Furthermore, Palm et al. demonstrated in RPE-1 cells that Ψ substitution of the PRINT donor RNA increased the proportion of productive insertions, consistent with reduced 5′ truncation. Improved RNA-template preservation may contribute to this effect, although the underlying mechanism remains to be established [17].

### 6.4. Adaptive Immune Responses

The absence of detectable innate immune activation in activated human T cells does not exclude adaptive immune responses to the encoded editing proteins. Non-human retrotransposon proteins, including those derived from R2, L2-2, Vingi-1, and RTE-1, may elicit antibody and T-cell responses following in vivo administration. For instance, pre-existing humoral and cellular immunity to SpCas9 and SaCas9 has been demonstrated in humans [68]. In mice previously immunized against SaCas9, sustained AAV-mediated expression of the editor led to immune-mediated loss of edited hepatocytes [69]. These findings highlight the importance of assessing all protein components of the editing machinery. Transient expression from RNA may limit antigen exposure but should not be assumed to prevent immune sensitization or immune-mediated clearance of cells expressing the editor.

Assessment could begin by testing clinically relevant RNA formulations in primary human target cells and peripheral blood mononuclear cells from multiple donors. Readouts should include viability, interferon responses, and cytokine and chemokine release, with appropriate controls to distinguish contributions from the RNA and delivery vehicle.

Protein-directed immunity should also be investigated by screening for pre-existing anti-editor antibodies and assessing antigen-specific T-cell responses across donors with diverse HLA backgrounds. Responses to individual retrotransposon proteins and accessory editors could be assessed using autologous antigen-presenting cells loaded with peptide pools or expressing the relevant proteins, with T-cell activation, proliferation, and cytokine production as readouts.

Studies in immunocompetent animals using the intended formulation and administration route should combine early cytokine profiling with longitudinal measurements of anti-editor antibodies, editor-specific CD4+ and CD8+ T-cell responses, tissue inflammation, and the persistence and function of edited cells. Repeat administration should be evaluated where relevant to the proposed therapeutic regimen.

Detection of anti-editor antibodies alone does not establish impaired editing or loss of edited cells. Immune responses should therefore be interpreted alongside editor-expression kinetics and therapeutic outcomes. Because immune recognition of editor proteins can differ between species, animal findings should be interpreted alongside the results obtained in human cell models.

### 6.5. Programmability

Achieving programmable integration currently requires a hybrid retrotransposon–nickase system. Among the platforms reviewed here, STITCHR and CREATE-RetroT offer the greatest flexibility in selecting genomic integration sites, but this flexibility increases both the number and size of the components that must be co-delivered, imposing additional formulation and stoichiometric constraints.

Previous attempts to retarget LINE-1 integration by fusing ORF2p to DNA-binding domains were unsuccessful [70], highlighting the difficulty of reprogramming this large multifunctional protein. Two alternative strategies have therefore emerged to maintain more compact and operationally simple systems. The first exploits the intrinsic target specificity of R2 proteins to integrate payloads into the multicopy 28S rDNA locus. The second uses APE-type elements such as Vingi-1 L2-2 and RTE-1 to distribute integrations broadly throughout the genome. The latter strategy lacks locus-level control but retains important advantages over lentiviral vectors by enabling the complete system to be formulated as RNA in LNPs for in vivo delivery while avoiding expensive and complex viral-vector manufacturing.

These approaches may be suitable for applications such as CAR expression or enzyme-replacement therapy, in which durable payload expression does not require regulation by a specific endogenous locus. Programmable platforms substantially broaden the potential applications by enabling whole-gene replacement and mutation-agnostic treatments capable of addressing multiple pathogenic variants within the same gene. They can also support precise insertion of promoterless payloads downstream of endogenous regulatory elements, thereby maintaining tissue-specific or physiologically regulated transgene expression while avoiding random integration of an exogenous promoter.

### 6.6. Clinical Application Considerations

The clinical opportunity for retrotransposon-based editing will likely depend on how well each platform architecture aligns with its intended therapeutic application. The optimal integration strategy will therefore vary by therapeutic indication (Table 2). Site-specific systems offer greater control over insertion location and may be favored when endogenous regulation, promoterless expression, or a defined genomic safe harbor is important, but they generally require additional targeting components and more complex co-delivery. Broadly integrating systems may be simpler and can achieve high levels of functional engineering, but their clinical development requires a substantially deeper understanding of insertion-site distribution, total and clonal copy numbers, incomplete products, and long-term clonal behavior. R2 systems occupy an intermediate position by exploiting a naturally preferred multicopy locus, although the consequences of repeated rDNA disruption and nuclease-associated DNA damage remain important considerations. These trade-offs should be evaluated in the context of the therapeutic threshold: applications requiring only a small fraction of durably modified cells may tolerate lower editing efficiency in exchange for greater genomic control, whereas cell-therapy applications may require higher editing levels but face greater concern regarding clonal expansion and insertional genotoxicity.

## 7. Conclusions

Retrotransposon-based platforms enable stable integration of multi-kilobase payloads from RNA templates without requiring an exogenous DNA donor, creating opportunities for nonviral in vivo genome engineering. The systems reviewed here encompass rDNA-targeted, broadly integrating, and programmable approaches, each with distinct advantages and limitations. Despite remaining technical challenges, these systems have rapidly progressed from mechanistic studies to preclinical demonstrations of durable gene integration in primary human cells and, for some platforms, in vivo. Nevertheless, they present distinct clinical trade-offs in editing efficiency, integration-site and copy-number control, payload integrity, delivery complexity, and genomic risk. Progress toward clinical translation will therefore depend on more than maximizing editing efficiency. It will require reproducible editing in disease-relevant cells, clinically meaningful functional outcomes, comprehensive characterization of integration products and insertion sites, and evidence of long-term safety in vivo. Addressing these requirements could establish retrotransposon-based platforms as an important complement to nuclease-, recombinase-, and viral-vector-based genome engineering, with platform selection guided by the requirements of each therapeutic application.

## Figures and Tables

**Figure 1:**
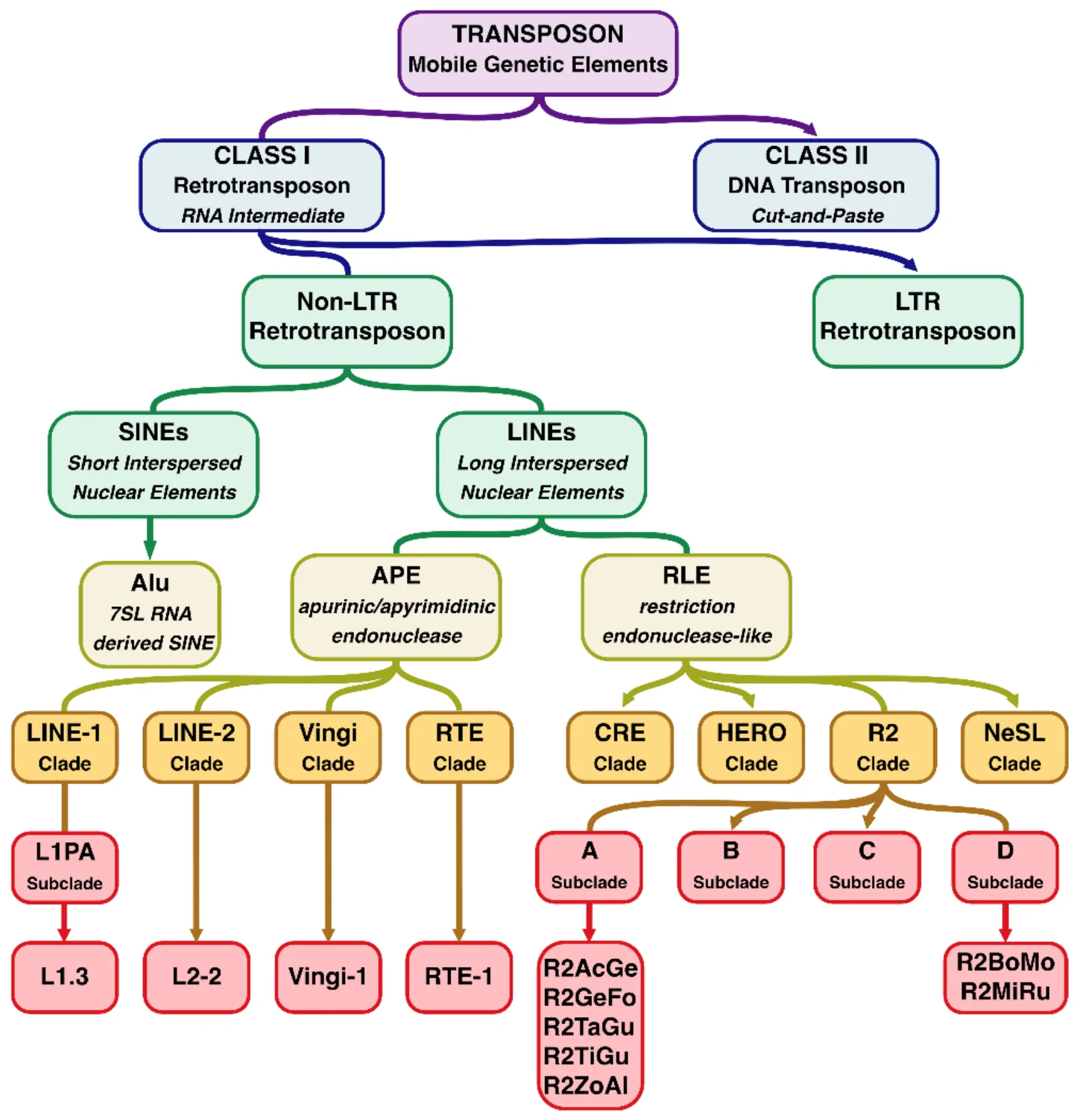
Classification of transposon clades. Mobile genetic elements are broadly divided into two classes according to whether their propagation involves an RNA intermediate. Among retrotransposons, LTR elements rely on an integrase to complete genomic insertion, while non-LTR (nLTR) retrotransposons integrate through target-primed reverse transcription (TPRT), in which reverse transcription occurs directly at the genomic site of insertion. Autonomous nLTR retrotransposons, including LINEs, can be further grouped according to the structure and evolutionary origin of their endonuclease domains into apurinic/apyrimidinic endonuclease (APE)- and restriction enzyme-like endonuclease (RLE)-containing lineages. Members of both groups are currently being repurposed as platforms for genome engineering and therapeutic gene insertion.

**Figure 2:**
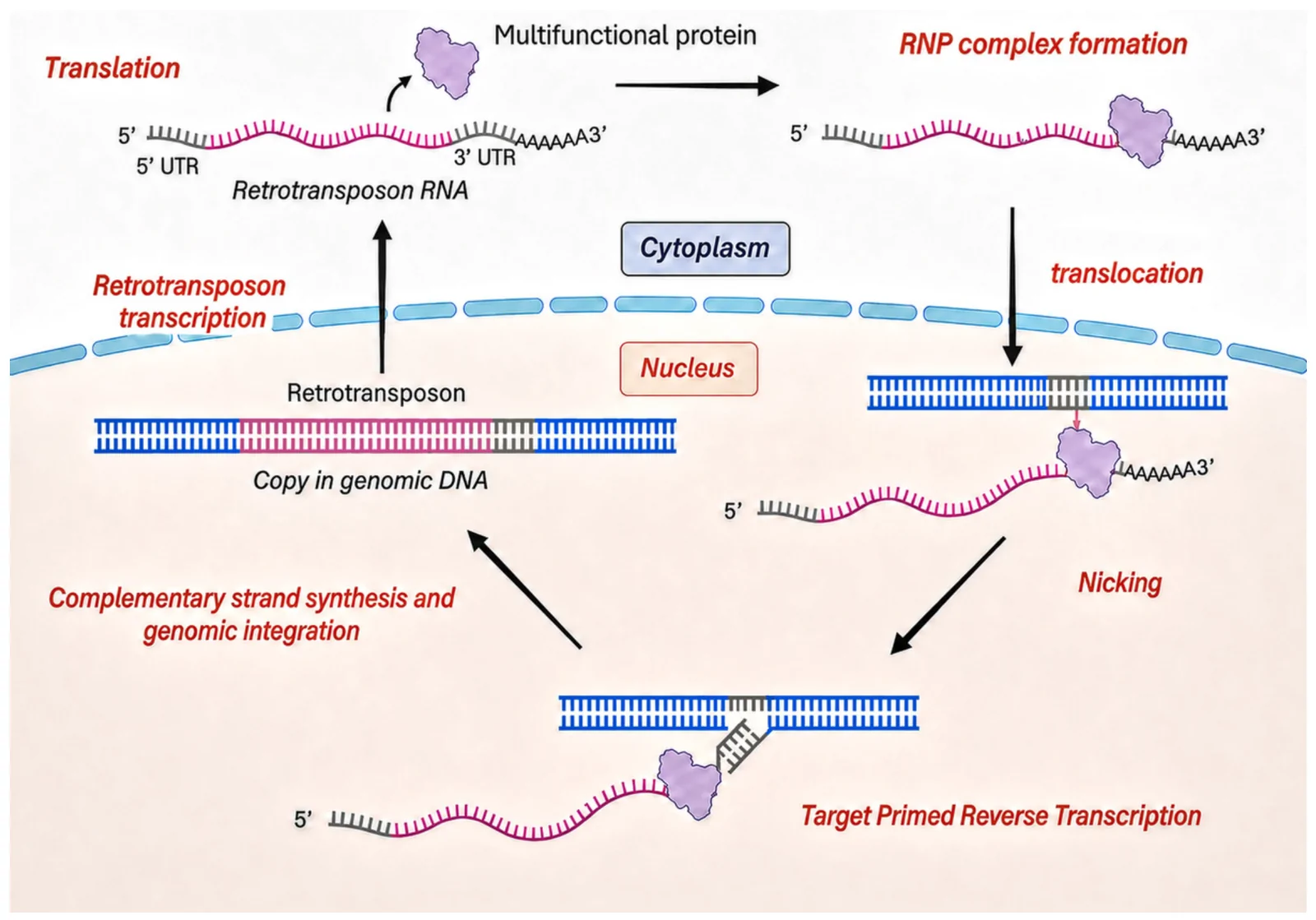
Retrotransposon life cycle. A genomic retrotransposon copy is transcribed into an RNA encoding a multifunctional protein containing endonuclease and reverse transcriptase domains. Following translation, the protein binds its encoding RNA to form a ribonucleoprotein (RNP) complex that translocates to the nucleus. At the genomic target site, the endonuclease nicks the bottom DNA strand, exposing a 3′ hydroxyl that primes reverse transcription of the retrotransposon RNA through target-primed reverse transcription. The cleavage of the top DNA strand exposes a second 3′ hydroxyl that primes complementary-strand synthesis. Host repair factors remove the displaced target DNA and complete integration of the newly synthesized retrotransposon copy.

**Figure 3:**
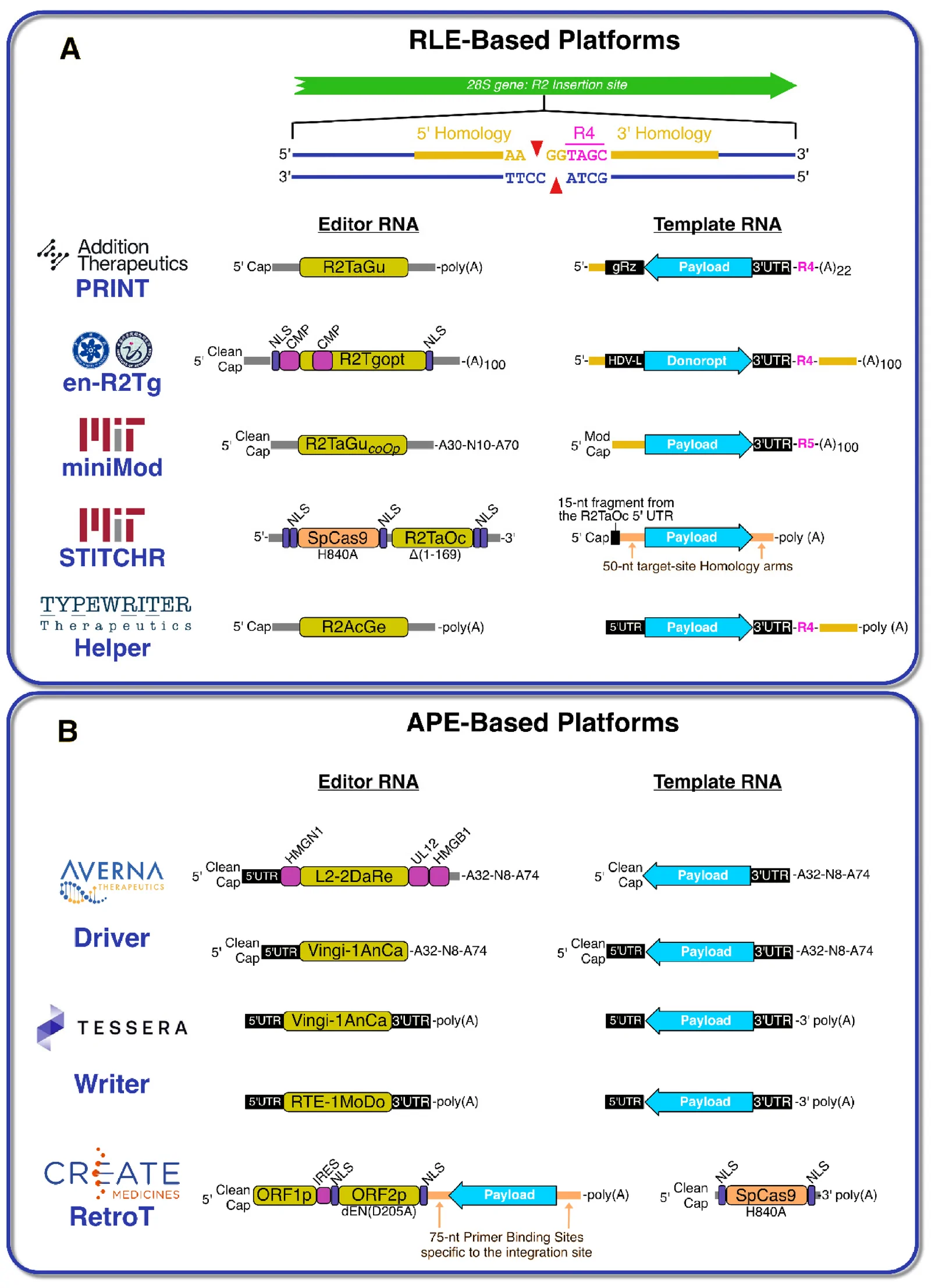
Retrotransposon-based platforms. Schematic representation of retrotransposon-based platforms with potential therapeutic applications. RNAs encoding the retrotransposon proteins are shown on the left, and payload-carrying RNAs are shown on the right. Because much of the available information on these platforms is scattered across patents, conference presentations, and other sources that have not undergone peer review, this figure represents our best effort to reconstruct their reported architectures. Retrotransposon UTRs are shown as white segments within black rectangles, whereas non-retrotransposon UTRs are shown in gray. Cap and poly(A)-tail structures are indicated when this information is available. (**A**) RLE-based platforms are built using R2 orthologs. The canonical R2 insertion site within the 28S rRNA gene is shown at the top, with nick positions indicated by red triangles. Homology to the sequence immediately downstream of the first nick is indicated in pink as RN, where N denotes the length of the homologous sequence in nucleotides. Longer regions of upstream and downstream homology to the 28S rRNA gene are shown in yellow. STITCHR target-site homology arms are shown in light orange and correspond to sequences flanking the selected genomic integration site. (**B**) APE-based retrotransposon platforms generally rely on random integration and do not harbor regions homologous to the host genome. RetroT is the exception, as it directs payload integration to the genomic site defined by nCas9-generated nicks.

**Table 1:** Retrotransposon nomenclature.

Retrotransposon	Species	Source Lineage	Type	Sponsor	Published as	Platform Name	Integration Site	Reference
R2AcGe	*Eurasian goshawk*	Avian	RLE	Typewriter Tx	R2Ag	Helper	28S rDNA	[27,28,29]
R2GeFo	*Geospiza fortis*	Avian	RLE	Addition Tx	R2GeFo	PRINT	28S rDNA	[26,30,31,32]
R2MiRu	*Micrastur ruficollis*	Avian	RLE	Typewriter Tx	R2MicRuf	Helper	28S rDNA	[27,28,29]
R2TaGu	*Taeniopygia guttata*	Avian	RLE	Addition Tx	R2TaGu	PRINT	28S rDNA	[26,30,31,32]
				IoZ	R2Tg	en-R2Tg	28S rDNA	[33]
				MIT	R2Tg	miniMod	28S rDNA	[15]
R2TaOc	*Talpa occidentalis*	Mammalian	RLE	MIT	R2Tocc	STITCHR	Programmable genomic loci	[34]
R2TiGu	*Tinamus guttatus*	Avian	RLE	Addition Tx	R2TiGu	PRINT	28S rDNA	[26,30,31,32]
R2ZoAl	*Zonotrichia albicollis*	Avian	RLE	Addition Tx	R2ZoAl	PRINT	28S rDNA	[26,30,31,32]
L1	*Homo sapiens*	Mammalian	APE	CREATE Medicine	L1.3	RetroT	Programmable genomic loci	[22,35]
L2-2DaRe	*Danio rerio*	Actinopterygian	APE	Averna Tx	ZfL2-2	Driver	Random	[36,37]
RTE-1MoDo	*Monodelphis domestica*	Mammalian	APE	Tessera Tx	RTE-1_MD	Writer	Random	[32,38,39]
Vingi-1AnCa	*Anolis carolinensis*	Reptilian	APE	Averna Tx	Vingi-1_Acar	Driver	Random	[36,37]
				Tessera Tx	Vingi-1_Acar	Writer	Random	[32,38,39]

APE: apurinic/apyrimidinic endonuclease; IoZ: Institute of Zoology, Chinese Academy of Sciences; MIT: Massachusetts Institute of Technology; rDNA: ribosomal DNA; RLE: restriction-like endonuclease, Tx: Therapeutics.

**Table 2:** Potential clinical applications and translational considerations for retrotransposon-based therapeutic platforms.

Clinical Application	Selection Criteria	Compatible Integration Strategy	Potential Advantages	Key Limitations/Risks	Critical Translational Evidence
In vivo Immune-cell engineering(e.g. CAR/TCR)	Platform selection depends on whether locus-specific regulation or concurrent gene disruption is required	Ectopic receptor expression: Genomic safe-harbor integration Broadly dispersed integration CAR/TCR integration at locus-specific regulation or concurrent gene disruption is required Programmable integration	Durable receptor expression following transient RNA delivery Potential to avoid ex vivo manufacturing Potential for repeatable, nonviral delivery	Requires efficient targeting of therapeutically relevant primary cells Insertional genotoxicity and clonal expansion are central concerns Lymphodepletion requirements, immune context, and edited-cell persistence may	Editing fraction and phenotype in primary cells Insertion-site distribution Full-length integration rate Copies per edited cell Tracking clonal expansion in vivo efficacy and persistence
Gene replacement and mutation-agnostic correction	Integration at a specific locus Replacement of large gene portion. Scar free integration. Preservation of the reading frame and/or correct splicing	Programmable integration	Programmable insertion can replace large coding sequences Address multiple pathogenic variants with a single payload. Preserve or restore endogenous regulation	Typically requires precise locus selection and endogenous regulation co-delivery complexity may reduce in vivo efficiency Incorrect or partial integration may have deleterious consequences.	On-target full-length integration Allelic structure Expression regulation Off-target integration Local structural variants Functional rescue.
Durable therapeutic protein production(e.g., enzyme/antibody)	Platform selection depends on the required payload expression level, regulation, and acceptable integration profile (copy number/truncated payload)	Sustained expression at therapeutically appropriate levels. Genomic safe-harbor integration Broadly dispersed integration Endogenous regulation of the payload Programmable integration	A relatively small number of durably edited cells may provide therapeutically meaningful systemic protein level Potential for single-dose treatment; Liver and muscle are accessible delivery targets.	Expression level must be predictable and durable Multicopy integration may cause toxicity or impair cell fitness Immune responses to editor or transgene may limit redosing.	Dose–expression relationship Tissue biodistribution Copy number Durability Protein immunogenicity Long-term histopathology

Platform selection depends on the target cell type and therapeutic objective. Additional considerations include cis/trans RNA architecture, component co-delivery requirements, full-length integration rate, copy-number distribution, and the available efficacy and safety evidence.

## Data Availability

No new data were created or analyzed in this study. Data sharing is not applicable to this article.

## Supplementary Materials

The following supporting information can be downloaded at: https://www.mdpi.com/article/10.3390/pharmaceutics18101316/s1, Table S1 [27,28,32,35,36,37,55,71].

## Abbreviations

The following abbreviations are used in this manuscript:

AAV	Adeno-Associated Virus
AcGe	Accipiter gentilis
AnCa	Anolis carolinensis
APE	apurinic/apyrimidinic endonuclease
BLI	Bioluminescence imaging
BoMo	Bombyx mori
CAR	Chimeric Antigen Receptor
CREATE	CRISPR-Enabled Autonomous Transposable Element
DaRe	Danio rerio
DBS	Double-Strand Break
ENT	ENndonuclease-Tuned
EPO	Erythropoietin
IRES	Internal Ribosome Entry Site
GeFo	Geospiza fortis
gRZ	genomic-strand ribozyme
LINE	Long Interspersed Nuclear Element
LNP	Lipid NanoParticle
LTR	Long Terminal Repeat
m1Ψ	N1-methylpseudouridine
MiRu	Micrastur ruficollis
MoDo	Monodelphis domestica
nt	nucleotide
ORF	Open Reading Frame
PBS	Primer-Binding Site
PRINT	Precise RNA-mediated INsertion of Transgenes
rDNA	ribosomal DNA
RLE	Restriction enzyme-Like Endonuclease
RNP	RiboNucleoProtein
SINE	Short Interspersed Nuclear Elements
STITCHR	Site-Specific Target-primed Insertion Through targeted CRISPR Homing of Retroelements
TaGu	Taeniopygia guttata
TaOc	Talpa occidentalis
TiGu	Tinamus guttatus
tLNP	cell-targeted Lipid NanoParticle
TRuC	T cell Receptor fusion Construct
TPRT	Target-Primed Reverse Transcription
Unk	Unknown
UTR	UnTranslated Region
ZoAl	Zonotrichia albicollis

## References

[1] Beam Therapeutics Inc (2024). A Phase 1/2 Dose-Exploration and Dose-Expansion Study to Evaluate the Safety and Efficacy of BEAM-302 in Adult Patients with Alpha-1 Antitrypsin Deficiency (AATD)-Associated Lung Disease and/or Liver Disease.

[2] Verve Therapeutics, Inc (2023). Open-Label, Phase 1b, Single Ascending Dose Study to Evaluate the Safety of VERVE-102 Administered to Patients with Heterozygous Familial Hypercholesterolemia or Premature Coronary Artery Disease Who Require Additional Lowering of Low-Density Lipoprotein Cholesterol.

[3] Verve Therapeutics, Inc (2024). A Phase 1b Single Ascending Dose Study to Evaluate the Safety of VERVE-201 in Patients with Refractory Hyperlipidemia.

[4] Beam Therapeutics Inc (2024). A Phase 1/2, Dose-Exploration Study to Evaluate the Safety and Efficacy of BEAM-301 in Patients with Glycogen Storage Disease Type Ia (GSDIa) Homozygous or Compound Heterozygous for the G6PC1 c.247C>T (p.R83C) Variant.

[5] Prime Medicine, Inc (2026). A Phase 1/2 Clinical Study to Evaluate Safety, Tolerability, Biological Activity, and Initial Efficacy of Prime Editing (PM577a) for the Treatment of Wilson Disease (WD) in Adult and Adolescent Participants with at Least One Allele Harboring the p.H1069Q Mutation in ATP7B.

[6] Intellia Therapeutics Inc (2020). Phase 1 Two-Part (Open-Label, Single Ascending Dose (Part 1) and Open-Label, Single Dose Expansion (Part 2)) Study to Evaluate Safety, Tolerability, Pharmacokinetics, and Pharmacodynamics of NTLA-2001 in Patients with Hereditary Transthyretin Amyloidosis with Polyneuropathy (ATTRv-PN) and Patients with Transthyretin Amyloidosis-Related Cardiomyopathy (ATTR-CM).

[7] Behr M., Zhou J., Xu B., Zhang H. (2021). In vivo delivery of CRISPR-Cas9 therapeutics: Progress and challenges. Acta Pharm. Sin. B.

[8] Regeneron Pharmaceuticals (2024). A Two-Part Open-Label Study of REGV131-LNP1265, A CRISPR/Cas9 Based Coagulation Factor IX Gene Insertion Therapy in Participants with Hemophilia B.

[9] iECURE, Inc (2023). A Phase 1/2/3 First-in-Human, Open-Label, Dose-Escalation Study to Evaluate the Safety and Efficacy of a Single Intravenous (IV) Administration of ECUR-506 in Males Less Than 9 Months of Age with Genetically Confirmed Neonatal Onset Ornithine Transcarbamylase (OTC) Deficiency.

[10] Yarnall M.T.N., Ioannidi E.I., Schmitt-Ulms C., Krajeski R.N., Lim J., Villiger L., Zhou W., Jiang K., Garushyants S.K., Roberts N. (2022). Drag-and-drop genome insertion of large sequences without double-strand DNA cleavage using CRISPR-directed integrases. Nat. Biotechnol..

[11] Pandey S., Gao X.D., Krasnow N.A., McElroy A., Tao Y.A., Duby J.E., Steinbeck B.J., McCreary J., Pierce S.E., Tolar J. (2024). Efficient site-specific integration of large genes in mammalian cells via continuously evolved recombinases and prime editing. Nat. Biomed. Eng..

[12] Fuller M.G.A., Foley M., Barrangou R. (2026). CRISPR-based technologies for large DNA insertions. Trends Biotechnol..

[13] Thawani A., Collins K., Nogales E. (2025). Structural and biochemical studies of mobile retrotransposon proteins in action. Curr. Opin. Struct. Biol..

[14] Dangerfield T.L., Zhou J., Neumeyer J., Eickbush T., Johnson K.A. (2026). Single-turnover kinetic analysis of non-long terminal repeat retrotransposition defines the pathway and rate constants leading to second-strand synthesis. Nucleic Acids Res..

[15] Edmonds K.K., Wilkinson M.E., Strebinger D., Chen H., Lash B., Schaefer C.C., Zhu S., Liu D., Zilberzwige-Tal S., Ladha A. (2025). Structure and biochemistry-guided engineering of an all-RNA system for DNA insertion with R2 retrotransposons. Nat. Commun..

[16] Ghanim G.E., Hu H., Boulanger J., Nguyen T.H.D. (2025). Structural mechanism of LINE-1 target-primed reverse transcription. Science.

[17] Palm S.M., Horton C.A., Zhang X., Collins K. (2024). Structure and sequence at an RNA template 5′ end influence insertion of transgenes by an R2 retrotransposon protein. RNA.

[18] Wilkinson M.E., Frangieh C.J., Macrae R.K., Zhang F. (2023). Structure of the R2 non-LTR retrotransposon initiating target-primed reverse transcription. Science.

[19] Thawani A., Florez Ariza A.J., Nogales E., Collins K. (2023). Template and target-site recognition by human LINE-1 in retrotransposition. Nature.

[20] Kapitonov V.V., Tempel S., Jurka J. (2009). Simple and fast classification of non-LTR retrotransposons based on phylogeny of their RT domain protein sequences. Gene.

[21] Luo S., Chen Q., Chen Y., Li W. (2025). Biology and utilization of R2 retrotransposons. RNA Biol..

[22] Wang Y., Lin R.-Z., Harris M., Lavayen B., Diwanji N., McCreedy B., Hofmeister R., Getts D. (2025). CRISPR-Enabled Autonomous Transposable Element (CREATE) for RNA-based gene editing and delivery. EMBO Rep..

[23] Kojima K.K. (2018). Human transposable elements in Repbase: Genomic footprints from fish to humans. Mob. DNA.

[24] Nefedova L., Kim A. (2017). Mechanisms of LTR-Retroelement Transposition: Lessons from Drosophila melanogaster. Viruses.

[25] Zahavi E.E., Fainzilber M. (2026). Reading the SINEs—functionality in noncoding RNA ‘cheap genes’. RNA Biol..

[26] Zhang X., Van Treeck B., Horton C.A., McIntyre J.J.R., Palm S.M., Shumate J.L., Collins K. (2024). Harnessing eukaryotic retroelement proteins for transgene insertion into human safe-harbor loci. Nat. Biotechnol..

[27] Fujiwara H., Heffner G., Zhou J.U.N., Kami A., Su Y.-T. (2025). New R2 Retrotransposons for Gene Writing. WO Patent.

[28] Heffner G., Zhou J.U.N., Fujiwara H., Endo K.E.I. (2025). R2 Retrotransposons for Gene Writing. WO Patent.

[29] Heffner G., Endo K.E.I., Zhou J.U.N. (2025). Repeat Dosing of Retrotransposons. WO Patent.

[30] Venkataramanan S. (2026). Compositions for Target Primed Reverse Transcription. WO Patent.

[31] Venkataramanan S., Wells Reno James N. (2025). RNA Tails for Target Primed Reverse Transcription. WO Patent.

[32] (2026). ASGCT 2026 Annual Meeting Abstracts. Mol. Ther..

[33] Chen Y., Luo S., Hu Y., Mao B., Wang X., Lu Z., Shan Q., Zhang J., Wang S., Feng G. (2024). All-RNA-mediated targeted gene integration in mammalian cells with rationally engineered R2 retrotransposons. Cell.

[34] Fell C.W., Villiger L., Lim J., Hiraizumi M., Tagliaferri D., Yarnall M.T.N., Lee A., Jiang K., Kayabolen A., Krajeski R.N. (2025). Reprogramming site-specific retrotransposon activity to new DNA sites. Nature.

[35] Ding J., Harris M., Cochran E., Ty T., Leary G., Rodriguez J., Chal J., Wang Y., Getts D., Hofmeister R. (2026). Abstract LB155: Durable anti-tumor immunity through In Vivo CAR T cell engineering using the RetroT all-RNA genome integration platform. Cancer Res..

[36] Bassan D., Alvarez Rodriguez R., Trudeau D., Ron M., Ben Shushan-Goleczki Y. (2025). Compositions and Methods for Modulating T Cells. WO Patent.

[37] Ben Shushan-Goleczki Y., Kowalsman N., Trudeau D. (2024). Recombinant Proteins Comprising Non-LTR Retrotransposon-Derived Polypeptides for Gene Delivery and Insertion. WO Patent.

[38] Sherrill-Mix S., Lewinski M.K., Famiglietti M., Bosque A., Malani N., Ocwieja K.E., Berry C.C., Looney D., Shan L., Agosto L.M. (2013). HIV latency and integration site placement in five cell-based models. Retrovirology.

[39] Ngan Kevin C.-H., Boucher Jeffrey I.A.N., Steinberg Barrett E. (2025). Modified UTRs for Retrotransposon Systems. WO Patent.

[40] Baldwin E.T., van Eeuwen T., Hoyos D., Zalevsky A., Tchesnokov E.P., Sánchez R., Miller B.D., Di Stefano L.H., Ruiz F.X., Hancock M. (2023). Structures, functions and adaptations of the human LINE-1 ORF2 protein. Nature.

[41] Kojima K.K., Fujiwara H. (2003). Cross-Genome Screening of Novel Sequence-Specific Non-LTR Retrotransposons: Various Multicopy RNA Genes and Microsatellites Are Selected as Targets. Mol. Biol. Evol..

[42] Kojima K.K., Kapitonov V.V., Jurka J. (2010). Recent Expansion of a New Ingi-Related Clade of Vingi non-LTR Retrotransposons in Hedgehogs. Mol. Biol. Evol..

[43] Ran F.A., Hsu P.D., Lin C.-Y., Gootenberg J.S., Konermann S., Trevino A.E., Scott D.A., Inoue A., Matoba S., Zhang Y. (2013). Double Nicking by RNA-Guided CRISPR Cas9 for Enhanced Genome Editing Specificity. Cell.

[44] Sil S., Keegan S., Ettefa F., Denes L.T., Boeke J.D., Holt L.J. (2023). Condensation of LINE-1 is critical for retrotransposition. eLife.

[45] McIntyre J.J.R., Horton C.A., Collins K. (2026). Different DNA repair pathways support intact or truncated insertions by R2 retrotransposon protein. Science.

[46] Liu N., Lee C.H., Swigut T., Grow E., Gu B., Bassik M.C., Wysocka J. (2018). Selective silencing of euchromatic L1s revealed by genome-wide screens for L1 regulators. Nature.

[47] Protasova M.S., Andreeva T.V., Rogaev E.I. (2021). Factors Regulating the Activity of LINE1 Retrotransposons. Genes.

[48] U.S. Food and Drug Administration (2024). Human Gene Therapy Products Incorporating Human Genome Editing: Guidance for Industry.

[49] Su Y., Nichuguti N., Kuroki-Kami A., Fujiwara H. (2019). Sequence-specific retrotransposition of 28S rDNA-specific LINE R2Ol in human cells. RNA.

[50] Fujiwara H. (2015). Site-specific non-LTR retrotransposons. Microbiol. Spectr..

[51] Kuroki-Kami A., Nichuguti N., Yatabe H., Mizuno S., Kawamura S., Fujiwara H. (2019). Targeted gene knockin in zebrafish using the 28S rDNA-specific non-LTR-retrotransposon R2Ol. Mob. DNA.

[52] Schmidt M., Hoffmann G., Wissler M., Lemke N., Müßig A., Glimm H., Williams D.A., Ragg S., Hesemann C.-U., von Kalle C. (2001). Detection and Direct Genomic Sequencing of Multiple Rare Unknown Flanking DNA in Highly Complex Samples. Hum. Gene Ther..

[53] Jin W., Yu C., Zhang Y., Cao C., Xia T., Song G., Cai Z., Xue Y., Zhu B., Xu R.M. (2025). Mechanism of DNA targeting by human LINE-1. Science.

[54] Baeuerle P.A., Ding J., Patel E., Thorausch N., Horton H., Gierut J., Scarfo I., Choudhary R., Kiner O., Krishnamurthy J. (2019). Synthetic TRuC receptors engaging the complete T cell receptor for potent anti-tumor response. Nat. Commun..

[55] Steinberg Barrett E., Cotta-Ramusino Cecilia Giovanna S., Rodriguez Jason J., De Laco A., Li D., Kim K., Chen P.-F., Bothmer Anne H., Mir A., Schiroli G. (2025). Compositions and Methods for Modulating a Genome in T Cells, Induced Pluripotent Stem Cells, and Respiratory Epithelial Cells. U.S. Patent.

[56] Ostertag E.M., Kazazian H.H. (2001). Biology of Mammalian L1 Retrotransposons. Annu. Rev. Genet..

[57] Babushok D.V., Ostertag E.M., Courtney C.E., Choi J.M., Kazazian H.H. (2006). L1 integration in a transgenic mouse model. Genome Res..

[58] Bibillo A., Eickbush T.H. (2004). End-to-End Template Jumping by the Reverse Transcriptase Encoded by the R2 Retrotransposon. J. Biol. Chem..

[59] George J.A., Burke W.D., Eickbush T.H. (1996). Analysis of the 5′ Junctions of R2 Insertions with the 28S Gene: Implications for Non-LTR Retrotransposition. Genetics.

[60] Eickbush M.T., Eickbush T.H. (2011). Retrotransposition of R2 elements in somatic nuclei during the early development of Drosophila. Mob. DNA.

[61] Symer D.E., Connelly C., Szak S.T., Caputo E.M., Cost G.J., Parmigiani G., Boeke J.D. (2002). Human L1 Retrotransposition Is Associated with Genetic Instability In Vivo. Cell.

[62] Zingler N., Willhoeft U., Brose H.-P., Schoder V., Jahns T., Hanschmann K.-M.O., Morrish T.A., Löwer J., Schumann G.G. (2005). Analysis of 5′ junctions of human LINE-1 and Alu retrotransposons suggests an alternative model for 5′-end attachment requiring microhomology-mediated end-joining. Genome Res..

[63] Bibiłło A., Eickbush T.H. (2002). The reverse transcriptase of the R2 non-LTR retrotransposon: Continuous synthesis of cDNA on non-continuous RNA templates. J. Mol. Biol..

[64] Kahwaji N., Kotzian N., Prinz J.M., Pu Y., Kath J., Picht S., Hiller A.L., Klaas A., Dunne C.M., Launspach M. (2026). MRNA-based CAR T cell engineering: Unmodified mRNA enables high CAR expression without innate immune activation in T cells. Mol. Ther. Nucleic Acids.

[65] Karikó K., Muramatsu H., Welsh F.A., Ludwig J., Kato H., Akira S., Weissman D. (2008). Incorporation of Pseudouridine Into mRNA Yields Superior Nonimmunogenic Vector with Increased Translational Capacity and Biological Stability. Mol. Ther..

[66] Svitkin Y.V., Cheng Y.M., Chakraborty T., Presnyak V., John M., Sonenberg N. (2017). N1-methyl-pseudouridine in mRNA enhances translation through eIF2α-dependent and independent mechanisms by increasing ribosome density. Nucleic Acids Res..

[67] Mauger D.M., Cabral B.J., Presnyak V., Su S.V., Reid D.W., Goodman B., Link K., Khatwani N., Reynders J., Moore M.J. (2019). MRNA structure regulates protein expression through changes in functional half-life. Proc. Natl. Acad. Sci. USA.

[68] Charlesworth C.T., Deshpande P.S., Dever D.P., Camarena J., Lemgart V.T., Cromer M.K., Vakulskas C.A., Collingwood M.A., Zhang L., Bode N.M. (2019). Identification of preexisting adaptive immunity to Cas9 proteins in humans. Nat. Med..

[69] Li A., Tanner M.R., Lee C.M., Hurley A.E., De Giorgi M., Jarrett K.E., Davis T.H., Doerfler A.M., Bao G., Beeton C. (2020). AAV-CRISPR Gene Editing Is Negated by Pre-existing Immunity to Cas9. Mol. Ther..

[70] Ade C.M., Derbes R.S., Wagstaff B.J., Linker S.B., White T.B., Deharo D., Belancio V.P., Ivics Z., Roy-Engel A.M. (2018). Evaluating different DNA binding domains to modulate L1 ORF2p-driven site-specific retrotransposition events in human cells. Gene.

[71] Bollhorst T., Upton H., Gomez Hernandez Elsa A., Agarwal S., Foquet L. (2026). Formulations for RNA-Mediated Insertion of Transgenes. WO Patent.

